# An Active Learning Approach with Uncertainty, Representativeness, and Diversity

**DOI:** 10.1155/2014/827586

**Published:** 2014-08-11

**Authors:** Tianxu He, Shukui Zhang, Jie Xin, Pengpeng Zhao, Jian Wu, Xuefeng Xian, Chunhua Li, Zhiming Cui

**Affiliations:** ^1^School of Computer Science and Technology, Soochow University, Suzhou 215006, China; ^2^State Key Lab. for Novel Software Technology, Nanjing University, Nanjing 210093, China; ^3^Suzhou Vocational University, Suzhou 215104, China

## Abstract

Big data from the Internet of Things may create big challenge for data classification. Most active learning approaches select either uncertain or representative unlabeled instances to query their labels. Although several active learning algorithms have been proposed to combine the two criteria for query selection, they are usually ad hoc in finding unlabeled instances that are both informative and representative and fail to take the diversity of instances into account. We address this challenge by presenting a new active learning framework which considers uncertainty, representativeness, and diversity creation. The proposed approach provides a systematic way for measuring and combining the uncertainty, representativeness, and diversity of an instance. Firstly, use instances' uncertainty and representativeness to constitute the most informative set. Then, use the kernel *k*-means clustering algorithm to filter the redundant samples and the resulting samples are queried for labels. Extensive experimental results show that the proposed approach outperforms several state-of-the-art active learning approaches.

## 1. Introduction

According to an IDC report, the global data volume in 2014 has reached 8.7 ZB and will reach 40 ZB. With storage and transmission expanding PB level and EB level, it is indicated that big data will play an important role as important resources. Many supervised learning algorithms have been largely used in classification tasks [1, 2]. For a classification problem, the performance of classifier depends heavily on the labeled sample set. However, obtaining the labeled samples is very difficult while the labeled samples are scarce. In order to reduce the cost of labeling, active learning methods have been adopted to control the labeling process. Active learning is an effective method to solve these problems, which select high information content unlabeled samples to be labeled by experts [3, 4]. Querying the most informative instances is probably the most popular approach for active learning. Therefore, the querying strategy naturally becomes a research hotspot of active learning algorithms.

There are numerous different query strategies that have been used to decide which instances are most informative. The strategies are generally divided into two categories. One is based on uncertainty sampling [5, 6], which considers samples' uncertainty as information content and selects the most uncertain samples for labeling. Although most uncertainty query selection strategies have a wide range of applications and achieve good results in many circumstances, they fail to take information in the large amount of unlabeled instances into account and are prone to query outliers. Another category overcomes the disadvantages of uncertainty sampling and considers the samples' uncertainty and representativeness [7, 8].

In general, heuristic methods have been proposed to balance between the uncertainty and the representativeness of the selected sample. They encourage the selection of cluster centers. However, no measure has been taken to avoid repeating labeling samples in the same cluster. Namely, all methods above did not consider redundancy between selected samples. Batch mode active learning methods will be affected by this problem. In order to accelerate the learning process, it is necessary to speed up the learning process by selecting more than one sample each iteration. So it needs to examine the diversity of the selected samples. To solve the above problems, we propose a novel active learning strategy that exploits information content measured by uncertainty, representativeness, and diversity of unlabeled instances. Samples selected for labeling are with high uncertainty and representativeness and little redundancy.

Our new query selection measure includes two steps. The first step is acquiring high information content samples set by combining uncertainty sampling and representativeness sampling. For the high informative samples set, we apply diversity sampling to get the final samples for labeling. The combination of the two terms is given in a general weighted product form and we use the kernel *k*-means clustering algorithm for diversity sampling. We conduct experiments on a few benchmark datasets and present promising results for the proposed active learning approach.

## 2. Related Work

A typical active learning framework assumes that there is a small set of labeled data *L* and a large pool *U* of unlabeled data available. Firstly, *L* is used to train the classifier *C*. Then, queries are selectively drawn from the pool, which is usually assumed to be closed. Typically, instances are queried in a greedy approach, according to an information measure used to estimate all instances in the pool, and labels for them are assigned by an expert. These new labeled samples are included into *L* and the classifier *C* is retrained. Querying loops continue for some predefined iterations or until a stop criterion is met.

A large number of active learning techniques have been introduced in the literature. Many methods employ an uncertainty sampling principle to select the unlabeled instance they are most hesitant to label. In [5], the most uncertain instance is taken as the one that has the largest entropy value on its probable labels. However, in multiclass problems, the entropy does not often well reflect the uncertainty of the sample. Some may have larger classification uncertainty than the ones whose entropy may be higher. For the above problem, Joshi et al. [6] proposed a more effective active learning sample selection criterion BvSB. This criterion considers the difference between the probability values of the two classes having the highest estimated probability value as a measure of uncertainty, which results in a better performance in practical applications. Another common sampling strategy is based on the reduction of version space, among which query-by-committee (QBC) algorithm is the most popular one. QBC algorithms train a committee of classifiers and choose the instance on which the committee members most disagree [9]. In essence, the QBC is also based on uncertainty sampling. One immediate problem is that these approaches select samples close to the classification boundary in that they only consider uncertainty of samples, which are prone to be outliers. In order to avoid labeling outlier samples, representativeness sampling is an effective solution and there are some studies for a combination of uncertainty and representative aspects. References [10, 11] employ the unlabeled data by using the prior density as weights for uncertainty measures. A similar framework is proposed in [8], which uses a cosine distance to measure samples' representativeness. Literature [12] proposed an adaptive active learning method and showed better performance. Although different prediction models have been employed in these methods, they all ignore samples' cluster information or diversity information. Therefore these methods have the drawback of repeatedly labeling samples in the same cluster, which has little help for improving accuracy. In this paper, we develop a new active learning method which utilizes uncertainty sampling, representativeness sampling, and diversity sampling.

## 3. Proposed Approach

In this section, we present a novel active learning method that combines the three sampling criteria. The proposed active learning method has four key components: an uncertainty measure, a representativeness measure, an information content measure, and a diversity measure. We will introduce each of them below.

### 3.1. Uncertainty Measure

Uncertainty sampling aims to choose the most uncertain instance to label. We employ the best-versus-second-best (BvSB) [6] approach, which considers the difference between the probability values of the two classes having the highest estimated probability value as a measure of uncertainty. Assume that our estimated probability distribution for a certain example is denoted by *P*. Probability value of the best class guess and the second best guess are, respectively. We obtain the BvSB measure and refer [6] for detained information:

(1) Uncertainty(xi) =BvSB=argmin⁡xi∈U(p(yBest ∣ xi)−p(ySecond−Best ∣ xi)).

### 3.2. Representativeness Measure

As mentioned earlier, uncertainty sampling may suffer from the problem of selecting outlier samples. In order to prevent selecting these samples, representativeness sampling is an effective solution. The representativeness of a sample can be evaluated based on how many samples there are similar to it. So, samples with high representativeness are less likely to be outliers. In this section, we use the Gaussian Process [13] framework to measure the representativeness information between the current sample and the remaining unlabeled sample set.

Similar to the literature [12], we define representativeness measure for a candidate sample *x*_*i*_ as follows:

(2) $$\begin{array}{c} \text{Rep}\left(x_{i}\right)=H\left(x_{i}\right)-H\left(x_{i}\;|\;U_{x_{i}}\right), \end{array}$$

where *U*_*x*_*i*__ denotes the set of unlabeled instances after removing *x*_*i*_ from *U* and *H*(*x*_*i*_) and *H*(*x*_*i*_∣*U*_*x*_*i*__), respectively, represent entropies of *x*_*i*_ and the remaining unlabeled samples.

A Gaussian Process is a joint distribution over a set of random variables and the marginal distribution over any finite subset of variables is multivariate Gaussian. So we compute the entropy terms with it. For our issue, each instance is associated with a random variable. A symmetric kernel function *K*(·, ·) is then used to produce the covariance matrix, such that

(3) $$\begin{array}{c} \sum_{ii}=K\left(x_{i},x_{i}\right), \end{array}$$

(4) $$\begin{array}{c} \sum_{U_{i}U_{i}}=\left(\begin{array}{cccc} K\left(x_{1},x_{1}\right) & K\left(x_{1},x_{2}\right) & \cdots & K\left(x_{1},x_{n}\right)\\ K\left(x_{2},x_{1}\right) & K\left(x_{2},x_{2}\right) & \cdots & K\left(x_{2},x_{n}\right)\\ \vdots & \vdots & \vdots & \vdots \\ K\left(x_{n},x_{1}\right) & K\left(x_{n},x_{2}\right) & \cdots & K\left(x_{n},x_{n}\right) \end{array}\right), \end{array}$$

where the covariance matrix ∑_*U*_*i*_*U*_*i*__ is actually a kernel matrix defined over all the unlabeled instances and we assume *U*_*i*_ = {1,2,…, *n*}.

According to the property of multivariate Gaussian distribution, we can know that

(5) $$\begin{array}{c} \sum_{i\;\;|\;\;U_{i}}^{2}=\sum_{ii}^{2}-\sum_{iU_{i}}\sum_{U_{i}U_{i}}^{-1}\sum_{U_{i}i}. \end{array}$$

Closed-form solutions exist for the entropy terms such that

(6) H(xi)=12ln⁡(2πe∑ii),H(xi ∣ Uxi)=12ln⁡(2πe∑i  ∣  Ui).

Using (6), the representativeness definition can finally be in the following form:

(7) $$\begin{array}{c} \text{Rep}\left(x_{i}\right)=\frac{1}{2}\ln\left(\frac{\sum_{ii}}{\sum_{i\;\;|\;\;U_{i}}}\right). \end{array}$$

### 3.3. Information Content Measure

Given the uncertainty measure and the representativeness measure defined above, we seek to combine the strengths of both. The main idea is to pick samples that are not only with high uncertainty but also with high representativeness. We use the combination value of the two measures as information content value. The higher the combination value is, the higher the information content of corresponding sample is.

Specifically, we propose to combine the two values in a general product form and the information content of sample *x*_*i*_ is as follows:

(8) $$\begin{array}{c} \text{Infor}\left(x_{i}\right)=\alpha *\text{Uncertainty}\;\;\left(x_{i}\right)*\text{Rep}\left(x_{i}\right), \end{array}$$

where *α* is a tradeoff controlling parameter over the two terms. Samples with high Infor(*x*_*i*_) value are more likely to be selected for labeling.

### 3.4. Diversity Measure

As we know, the high information content set may contain samples in the same cluster. In order to avoid selecting superfluous samples, we apply the kernel *k*-means clustering algorithm to cluster samples with high information content. We get the *k* clusters *C*_1_, *C*_2_,…, *C*_*k*_.

Then we choose the *k* cluster centers *S*_*k*_ = {*x*_*C*_1__, *x*_*C*_2__,…, *x*_*C*_*k*__} for labeling, which can effectively guarantee that samples for labeling are with high information content and little redundancy.

First we consider representativeness and diversity criteria at the same time and get the high information content set *S*. Furthermore, diversity sampling is considered and redundant samples are filtered. So we cluster the samples in the high information content set and choose the clustering center of each cluster into a batch for labeling.

The overall framework of our active learning algorithm is given in Algorithm 1.

## 4. Experimental Results

In order to evaluate the effectiveness of our proposed approach described in previous sections, we demonstrate results on three UCI datasets: the Letter Recognition Data Set, USPS: optical recognition of handwritten digits originally from the US Postal Service, and Pendigits: pen-based recognition of handwritten digits. The chosen datasets and their properties are summarized in Table 1 along with initial samples set, unlabeled samples set, and test set sizes used in our experiments.

The experiments are conducted to compare the proposed active learning approach to a number of active learning methods, including (1) BvSB [6], which is the uncertainty sampling method, and (2) information density (ID), which denotes the active learning method in [8] that uses the cosine distance to measure an information density and selects uncertain and representative instances.

LibSVM is employed to train the train a SVM classifier for all these approaches, and it provides probabilistic predictions over the class labels.

### 4.1. Size of *k* for Each Dataset

Table 2 shows the number of cluster for each dataset. Note that class numbers of each dataset have already been known in advance. Then, we adjust the *k* parameter according to the class number of each dataset to cluster current high information content samples set.

### 4.2. Classification Accuracy on Three Datasets

**Figure 1:**
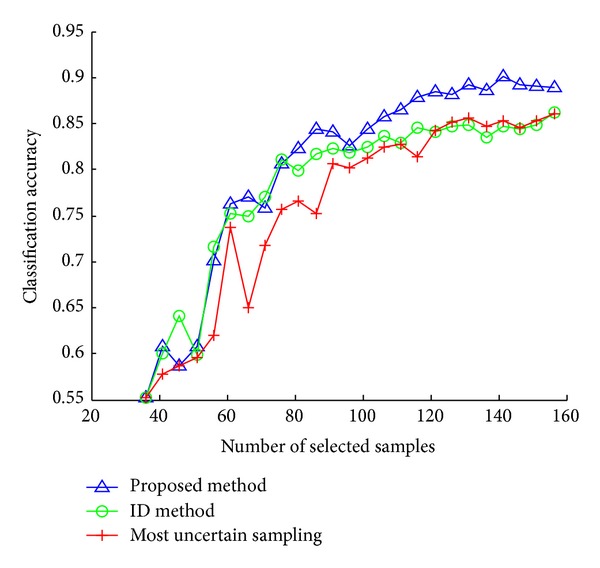
Results on USPS dataset.

In Figure 1, we show results on the USPS dataset, a dataset consisting of handwritten digits from the US Postal Service. At each active learning round, we select 10 samples for labeling. At early iterations, performances of all methods are similar. As the number of labeled samples increases, our method gradually dominates the other two approaches and the proposed approach selects the most useful samples.

This difference between the three active selection methods becomes more clearly when we look at the results on the Letters dataset. Similar to USPS dataset, we select 10 samples for labeling. We can know that, for achieving the same value of classification accuracy on the test data, our method needs far fewer training samples than the other two approaches. Note that ID method does marginally better than most uncertain sampling. The difference can be attributed to the fact that ID method combines uncertainty and representativeness of samples while most uncertain sampling only considers uncertainty of samples.

Figure 3 shows classification accuracy plots on the Letters dataset, which has 26 classes. Most uncertain sampling and ID method perform even worse on this problem due to the larger number of classes. They give a bad indicator of information content of unlabeled samples in this case, and they give comparable poor performance. Even with a larger number of classes, the figure indicates that our approach outperforms other active selection methods.

### 4.3. Comparison of Diversity

**Figure 2:**
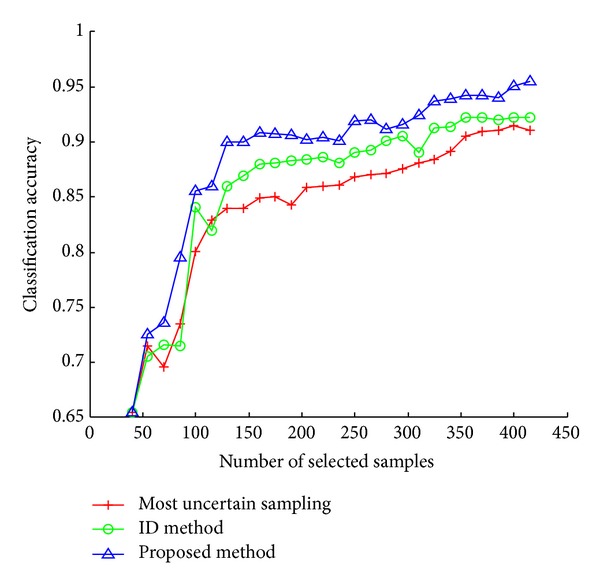
Results on Pendigits dataset.

**Figure 3:**
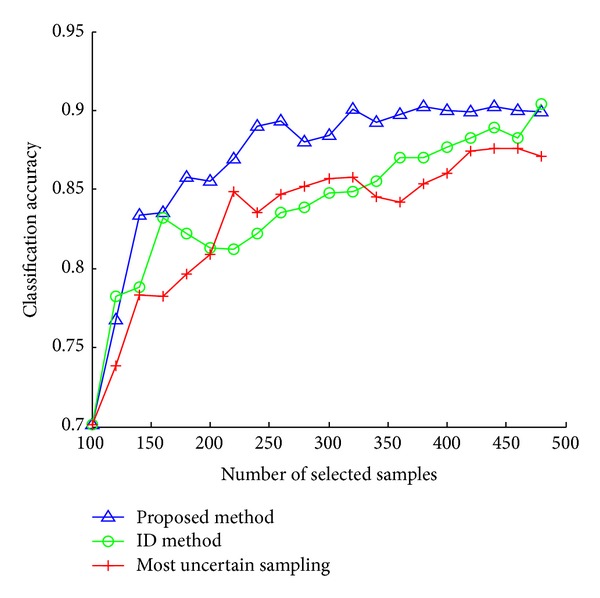
Results on Letters dataset.

**Figure 4:**
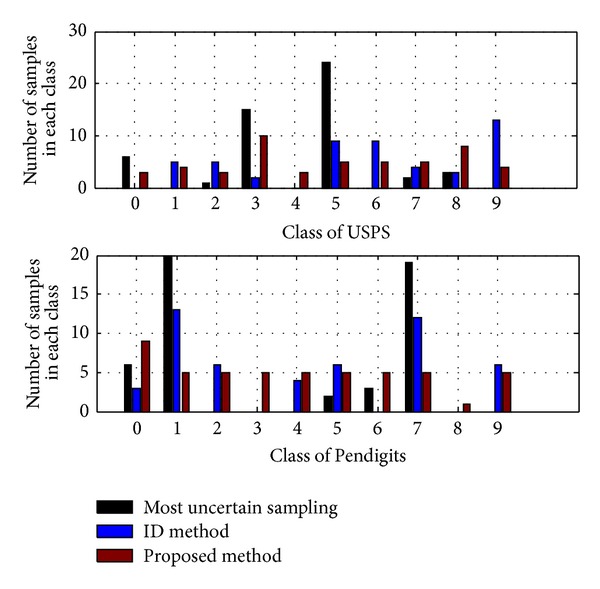
Comparison of diversity on 10 classes.

**Figure 5:**
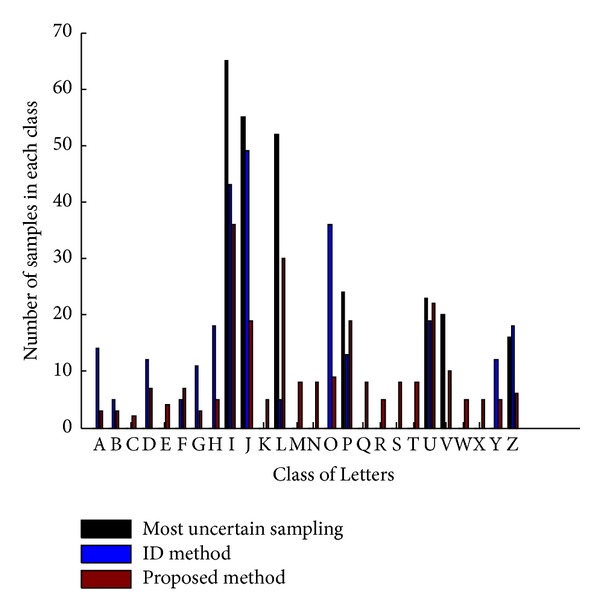
Comparison of diversity on 26 classes.

All the results show that our approach in selecting diversity samples is very effective, especially in Pendigits dataset (see Figure 2). From Figures 4 and 5, our method selects samples included in all classes while the other two methods only choose samples with uncertainty or representativeness which is distributed in only a part of classes. Therefore, the proposed method performs better than other methods in most cases.

## 5. Conclusion and Future Work

In this paper, we presented a novel adaptive active learning approach which combines uncertainty measure and representativeness measure with diversity measure together to conduct samples selection. The proposed method can select samples with high information content and little redundancy. Experiments on multiple datasets show advantages of our approach. The expert in our approach is assumed to be accurate, indefatigable (always answers the queries), and insensitive to costs. Labeling an optimal utility subset is still costly and expensive in many cases. Crowdsourcing labelers, which are composed of some cheap and noisy labelers, have now been considered for active learning. Future work will extend to reduce the cost of this issue.

## Figures and Tables

**Algorithm 1:**
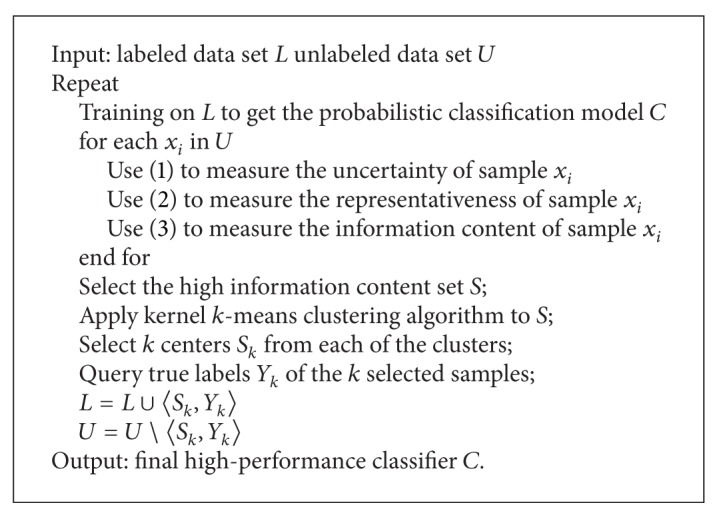
Incorporating uncertainty, representativeness, and diversity for active learning.

**Table 1:** Dataset properties and the corresponding sizes used.

Dataset	Classes	Features	Initial set size	Unlabeled set size	Test set size
USPS	10	256	30	5000	2000
Letters	26	16	30	5000	3000
Pendigits	10	16	30	7000	3498

**Table 2:** Cluster number for each dataset.

Dataset	Labeled numbers at each round
USPS	10
Pendigits	10
Letters	26
